# Delivery of canine rabies vaccination programme in Kutupalong-Balukhali refugee camps, Cox’s Bazar, Bangladesh

**DOI:** 10.1371/journal.pntd.0014143

**Published:** 2026-03-27

**Authors:** Luke Gamble, Karlette A. Fernandes, Keiichiro Tazawa, Rubaiya Ahmad, Kamrul Islam, James Hood, Bernadette Abela, Balaji Chandrashekar, Frederic Lohr, Ryan M. Wallace, Tim Parkin, Andrew D. Gibson, Catherine Swedberg

**Affiliations:** 1 Worldwide Veterinary Service, Cranborne, United Kingdom; 2 Obhoyaronno Animal Welfare Foundation, Dhaka, Bangladesh; 3 Directorate General of Health Services, Ministry of Health and Family Welfare, Dhaka, Bangladesh; 4 Department of Neglected Tropical Diseases, World Health Organization, Geneva, Switzerland; 5 Poxvirus and Rabies Branch, US Centers for Disease Control and Prevention, Atlanta, GeorgiaUnited States of America; 6 Bristol Veterinary School, University of Bristol, Langford, United Kingdom; Colorado State University, UNITED STATES OF AMERICA

## Abstract

**Background:**

Rabies causes over 60,000 deaths annually, primarily among children, with dog bites responsible for nearly all human cases. Although mass dog vaccination is effective in low-resource settings, structured campaigns have rarely been implemented in refugee camps, where unmanaged dog populations and limited access to post-exposure prophylaxis heighten rabies risk. This study aimed to demonstrate the possibility of delivering systematic mass dog vaccination within a short operational timeframe in a humanitarian setting and to assess community perceptions of rabies risk and prevention.

**Methodology/principal findings:**

In May 2025, a four-day mass dog vaccination campaign was conducted across the Kutupalong-Balukhali refugee settlement in Cox’s Bazar, Bangladesh, with real-time data collection to guide operations. Post-vaccination dog sight surveys assessed operational coverage, while community surveys evaluated knowledge, attitudes and practices (KAP) regarding rabies. Of the 2,275 dogs encountered, 1,781 (78.3%) were vaccinated, with 86.4% classified as unowned community dogs. The overall proportion of marked dogs across all surveyed zones was 71.5% (95% CI: 66.8–75.9%). In the community survey, 34.6% of 1,311 adult respondents (gender-adjusted) had heard of rabies, and 41.2% correctly identified dog bites as the primary route of transmission. Regarding appropriate post-bite care, 25.9% knew to both wash the wound and seek medical care. Approximately 8.7% of households experienced a dog bite in the preceding year, corresponding to a minimum annual incidence of 13.3 bites per 1,000 persons, equating to over 15,000 bites per year.

**Conclusions/significance:**

This study represents the first structured mass dog vaccination campaign in a refugee setting, demonstrating that rabies control can be effectively implemented even in complex humanitarian contexts. The campaign’s success offers a replicable model for integrating zoonotic disease prevention into emergency health responses, underscoring the critical need to safeguard displaced populations from preventable diseases.

## Author summary

Rabies is a deadly but preventable disease that kills more than 60,000 people each year, mostly children. Nearly all human infections are caused by bites from infected dogs. Vaccinating dogs is the most effective way to stop rabies from spreading, yet organized vaccination campaigns are rarely conducted in refugee settings, where free-roaming dogs are common and access to timely medical care after bites may be limited. We carried out a four-day mass dog vaccination campaign in the Kutupalong-Balukhali refugee settlement in Cox’s Bazar, Bangladesh. Using real-time data to guide field activities, we vaccinated over 78% of the dogs encountered during the campaign, most of which were unowned community dogs. We also surveyed residents to assess their knowledge of rabies and their experiences with dog bites. Awareness of rabies and appropriate wound care was limited, and dog bites were frequently reported. Our findings demonstrate that mass dog vaccination can be delivered in complex humanitarian settings and integrated into emergency health responses to reduce the risk of a preventable and fatal disease.

## Introduction

Rabies, a deadly but preventable neurotropic disease, causes an estimated 60,000 human deaths annually, with the highest burden in Asia and Africa [1]. Transmitted predominantly through the bite of an infected dog, rabies is invariably fatal once clinical symptoms appear, making it one of the most lethal zoonotic diseases [2]. Although effective vaccines exist for both humans and animals, dog-mediated rabies persists in many low- and middle-income countries (LMICs), driven by insufficient vaccination coverage among free-roaming dogs, limited access to post-exposure prophylaxis (PEP), and low healthcare seeking following bites [3]. Sustained mass dog vaccination achieving at least 70% coverage is recognised as the most effective strategy for interrupting dog-to-dog transmission and preventing human rabies deaths [2,4].

The burden of rabies falls disproportionately on vulnerable populations, particularly children [1,5]. Among these are individuals displaced by conflict or natural disasters and residing in refugee camps, where health services and infrastructure are often minimal or absent [6]. In such humanitarian settings, public health interventions typically prioritise acute conditions such as malnutrition, maternal health, and trauma care, while neglected zoonotic diseases such as rabies receive comparatively little attention [7,8]. Yet, excluding rabies from humanitarian health responses overlooks its long-term impacts, including preventable mortality, psychological trauma, and erosion of trust in health systems, particularly in settings where PEP is unavailable or inaccessible [9,10].

In Bangladesh, dog-mediated rabies remains endemic. Before the national rabies control programme was launched in 2011, thousands of human rabies deaths were estimated to occur each year [11,12]. Subsequent reductions in human cases have been attributed to scale-up of mass dog vaccination, expanded access to PEP, and alignment with the “*Zero by 30*” global elimination strategy developed by the World Health Organization (WHO) and partners [13–15]. However, the COVID-19 pandemic disrupted progress, pausing vaccination campaigns, weakening surveillance, and straining PEP supply chains [16]. As of 2025, sustaining ≥70% vaccination coverage in dog populations, strengthening public awareness, and ensuring consistent reporting remain ongoing challenges [13].

These constraints are amplified in humanitarian settings such as the Kutupalong-Balukhali refugee settlement in Cox’s Bazar District. Established in 2017 following the displacement of over 750,000 Rohingya people from Myanmar, the settlement now hosts more than one million residents and is one of the largest refugee settlements in the world [17]. Unlike many refugee contexts, there is no formal restriction on dog ownership or movement. Free-roaming, unvaccinated dogs are widespread, and permeable camp boundaries enable movement between refugee and host communities. In the absence of routine veterinary services, this unmanaged dog population, combined with frequent human–animal interactions, creates conditions conducive to rabies transmission.

A 2017 household survey reported that 6.4% of Kutupalong households experienced at least one dog bite that year, with a 5% fatality rate potentially attributable to rabies or secondary infection [18]. WHO Cox’s Bazar situation reports from 2021 to 2023 documented a substantial increase in reported dog bites, with frontline healthcare workers and Rohingya community leaders (*mahjis*) expressing growing concern, particularly regarding bites among children [19]. In July 2024, WHO distributed emergency PEP supplies in response to rising bite reports [20]. Although no laboratory-confirmed human or animal rabies cases have been reported within the settlement to date, limited diagnostic capacity and the absence of formal surveillance systems make under-detection likely. Despite these warning signals, no structured mass dog vaccination campaign had been conducted in the camp prior to May 2025 [21].

More broadly, despite increasing recognition of zoonotic risks in humanitarian settings, veterinary public health interventions remain rare or non-existent. To our knowledge, no structured canine vaccination programme has previously been implemented in a refugee camp, highlighting a critical gap and an opportunity for scalable One Health strategies in humanitarian responses.

In May 2025, the Government of Bangladesh, in partnership with Worldwide Veterinary Service (WVS) through its Mission Rabies programme and Obhoyaronno Bangladesh Animal Welfare Foundation, implemented the first mass dog vaccination campaign in the Kutupalong-Balukhali refugee settlement. This study describes the adaptation of established operational strategies and technologies, previously used in numerous LMICs, for application in a complex humanitarian context [22–24]. The primary objective was to assess the possibility of delivering mass dog vaccination within a short operational timeframe in a refugee settlement. A secondary objective was to assess community perceptions of rabies risk and prevention practices.

## Methods

### Ethics statement

This study protocol was developed in collaboration with Bangladesh’s Directorate General of Health Services (DGHS) and Department of Livestock Services (DLS), and was approved by the United States Centers for Disease Control Human Research Protection Office (Protocol ID 060118JB). All animals were handled by trained staff. Informed verbal consent was obtained from all participants prior to survey administration, and no personally identifiable information was collected.

### Study site

**Fig 1:**
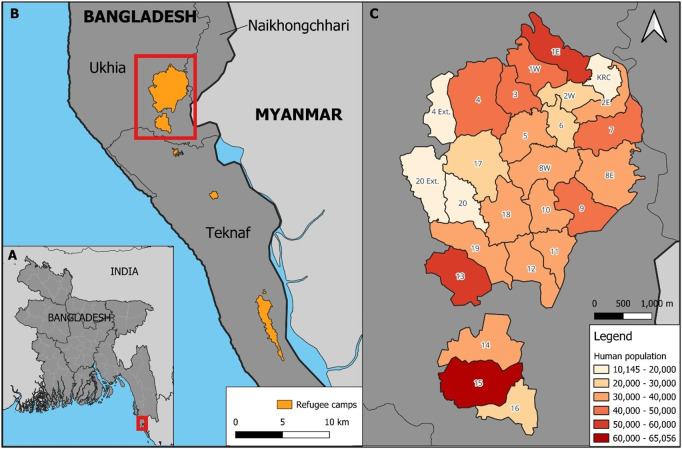
Maps of study area. **(A)** Map of Bangladesh showing administrative divisions (black borders) and districts (gray borders). **(B)** Location of refugee camps in Cox’s Bazar District (orange), with sub-district boundaries shown in black. **(C)** Kutupalong-Balukhali refugee settlement, showing sub-camp boundaries (black), and shaded by human population from 2025 census data [25]. Polygon and line data were obtained from Humanitarian Data Exchange [26].

In May 2025, a mass dog vaccination campaign against rabies was conducted in the Kutupalong-Balukhali refugee camps and surrounding areas, located in the Ukhiya sub-district, Cox’s Bazar District, Chattogram Division, Bangladesh (**Fig 1**). At the time of the study, the estimated population residing within camp boundaries was 1,139,433 people, distributed across 235,878 households, with a mean household size of 4.8 persons [25]. The camps span an area of approximately 18.35 square kilometres (km^2^), corresponding to an estimated population density of 62,087 persons per km^2^. Children under 18 years of age comprised 51.6% of the population (n = 587,989). Of the total population, 587,291 residents were female (51.5%) and 552,142 (48.5%) were male [25].

### Campaign logistics

Campaign operations were led by Bangladesh’s DGHS in partnership with Obhoyaronno Bangladesh Animal Welfare Foundation, with technical and logistical support from WVS through its Mission Rabies programme. Formal authorisation was granted by the Ministry of Health and Family Welfare, the Ministry of Home Affairs, and the Office of the Refugee Relief and Repatriation Commissioner (RRRC). Camp access and field coordination were facilitated by RRRC officials at designated Camp-in-Charge (CiC) offices.

Coordination among national and international stakeholders was conducted via shared digital platforms. Field guidance and security briefings were delivered by WHO, Médecins Sans Frontières (MSF), and the United Nations Children’s Fund (UNICEF).

An open call attracted over 400 applications from final-year veterinary students, interns, and recent graduates in Bangladesh, of whom 92 were selected. Field teams also included 25 trained animal handlers from DGHS and 40 community engagement volunteers. In accordance with WHO occupational health guidelines for rabies prevention [2], all personnel were required to provide documentation of rabies pre-exposure prophylaxis (PrEP) prior to campaign activities.

### Dog vaccination campaign

The vaccination campaign was conducted over four consecutive days across all 26 sub-camps of Kutupalong-Balukhali and surrounding areas, with field operations from 09:00–15:00 daily. Twenty-six vaccination teams (one per sub-camp) were deployed, each comprising four to five members: one vaccinator, one data collector, one dog handler, one assistant, and one logistics coordinator. A trained net-catching unit supported vaccination of difficult-to-handle dogs, particularly along camp peripheries. Rohingya mahjis and camp volunteers assisted teams with navigation and community engagement.

Sub-camp boundaries were subdivided into 144 distinct working zones following a previously described microplanning framework [22]. Zones were allocated using the WVS digital platform, with maps preloaded onto the WVS App to enable offline navigation in areas with limited network connectivity.

Each dog underwent a brief behavioural and health assessment. Dogs deemed clinically fit received a 1 mL dose of rabies vaccine (Nobivac Rabies, MSD Animal Health), administered subcutaneously or intramuscularly according to manufacturer instructions. Puppies under three months of age were eligible for vaccination. Vaccinated dogs were temporarily marked with non-toxic paint for visual identification and to avoid repeat vaccination during the campaign.

Vaccination data were recorded in real time using the WVS App. For each dog encountered, teams recorded sex, estimated age category, general health status, lactation status (for females), and ownership status. Dogs were classified as ‘unowned’ if free-roaming and unclaimed during team encounters, and as ‘owned’ if visibly associated with a household or physically restrained. For unvaccinated dogs, the primary reason for non-vaccination was recorded. GPS coordinates and timestamps were automatically captured at the point of data entry.

### Dog sight surveys

Post-vaccination sight surveys were conducted in randomly selected working zones where vaccination had been completed on the previous day. Six survey teams, each comprising two surveyors, walked zigzag transects throughout their assigned zones, recording all dogs sighted using the WVS App, following previously described methods [24]. Surveyors were independent of the vaccination teams to reduce bias related to knowledge of the locations of campaign activities.

For each dog, surveyors recorded sex, estimated age category, lactation status (for females), neuter status, and presence of a vaccination paint mark. The proportion of marked dogs among those sighted was used as a rapid operational indicator of coverage. Zones with ≥70% marked dogs were considered operationally complete, whereas zones with <70% marked dogs were revisited for mop-up vaccination, time permitting.

### Rabies awareness and community sensitisation

Forty community engagement volunteers, selected based on prior public health messaging experience within Kutupalong-Balukhali and fluency in Chittagonian, conducted activities beginning one day prior to vaccination and continuing throughout the four-day campaign. All volunteers received training on rabies prevention, safe dog handling, and safeguarding protocols.

Sensitisation teams were deployed ahead of vaccination activities using a coordinated block-based approach. Rabies awareness messages and information about the campaign were delivered through loudspeaker announcements, banners, posters, and leaflets. Age-appropriate rabies education lessons were delivered in learning centres, and wristbands were distributed to promote awareness. All Information, Education, and Communication (IEC) materials were reviewed for scientific accuracy and aligned with United Nations High Commissioner for Refugees (UNHCR) and WHO guidance for public health communication in humanitarian settings [27].

### Community survey

Community surveys were conducted concurrently with sensitisation activities among randomly selected adult residents (≥18 years) across all 26 sub-camps. While children, who are disproportionately affected by rabies, were not directly interviewed due to ethical considerations, adult respondents provided information on household members, including children, to capture bite exposures at the household level. A brief, structured questionnaire with Chittagonian translation was used for face-to-face interviews. Knowledge, attitudes, and practices (KAP) survey questions collected demographic data and assessed rabies awareness, perceived risk, bite response behaviours, and attitudes toward the vaccination campaign. Data were collected digitally in real time using the WVS App by trained personnel who were independent of vaccination teams to minimise bias.

### Data analysis

Descriptive analyses were performed using RStudio (version 4.3.1). Summary statistics were calculated to assess vaccination coverage, operational performance, and bite incidence. Vaccination coverage was estimated as the proportion of dogs sighted with a paint mark, with 95% confidence intervals (CIs) calculated assuming a binomial distribution. Post-vaccination dog sight survey data were summarised to evaluate operational coverage.

Community survey responses were summarised and adjusted for respondent gender using post-stratification weights to improve representativeness. Weights were calculated as the ratio of the adult (>18 years) camp population gender distribution (female 54%, male 46%) [25] to the observed sample proportions and applied to all summary estimates.

Annual dog bite incidence was estimated from the proportion of households reporting at least one bite among members in the preceding 12 months. As multiple bites per household were not recorded, one bite per reporting household was assumed, providing a minimum estimate. Person-level incidence per 1,000 population was calculated by dividing the household-level proportion by the mean survey household size and multiplying by 1,000, with associated 95% binomial CIs.

Summary figures were generated in R, with spatial visualisations of vaccination data created using the *sf* and *ggplot2* packages. Campaign location maps were prepared in QGIS Desktop (version LTR 3.40).

## Results

### Dog vaccination campaign

**Fig 2:**
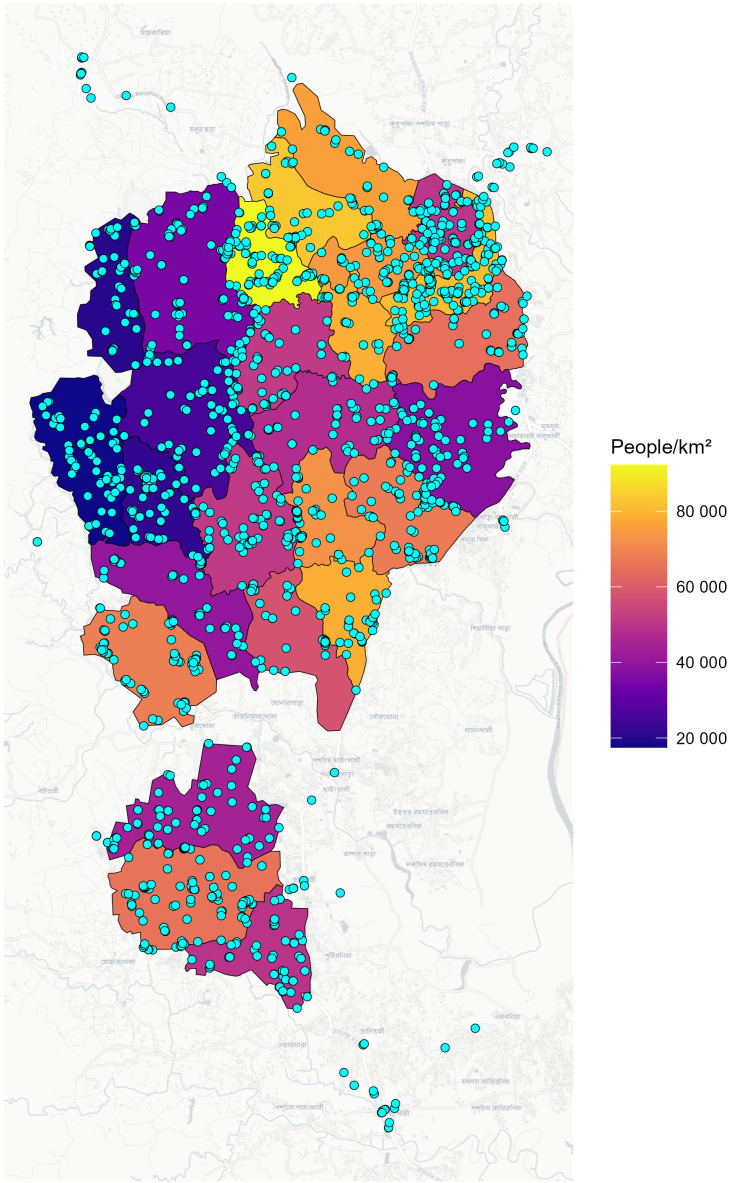
Location of dogs vaccinated during the 26-29 May 2025 campaign in Kutupalong-Balukhali. Map of 26 sub-camps in Kutupalong-Balukhali (black borders), coloured by human population density. Blue points indicate the GPS locations of individual vaccinated dogs recorded during the campaign. Administrative boundaries were obtained from Humanitarian Data Exchange [26]. Basemap layers were provided by CARTO, with underlying geographic data from OpenStreetMap.

Between 26 and 29 May 2025, a total of 2,275 dogs were encountered during a four-day mass dog vaccination campaign in Kutupalong-Balukhali (S1 Data). Of these, 1,781 (78.3%) were successfully vaccinated against rabies (**Fig 2**). Most vaccinated dogs were located within formal camp boundaries (n = 1,680; 94.3%), while 101 (5.7%) were in peripheral areas adjacent to the settlement. The number of dogs vaccinated varied across sub-camps, with the highest totals recorded in Camp 02E (n = 119) and the Kutupalong Registered Camp (KRC; n = 103). Vaccination was primarily carried out by standard hand-catching teams, which administered 1,715 doses (96.3%), while a trained net-catching unit vaccinated 66 dogs (3.7%) that were difficult to handle.

**Fig 3:**
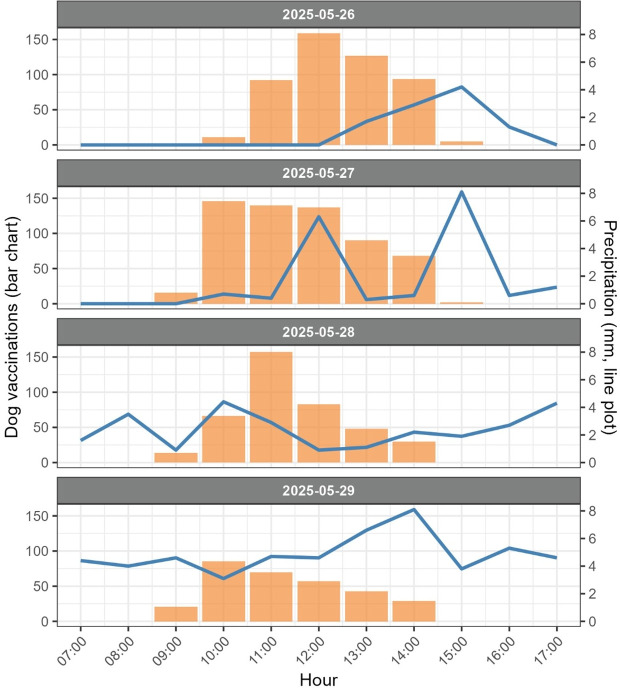
Hourly vaccination and precipitation levels. Hourly counts of vaccinations (bars, left y-axis) and precipitation in millimetres (line, right y-axis) from Meteostat.net. The x-axis shows the hour of the day (07:00-17:00). Bars represent the number of vaccinations administered, and the line represents total precipitation recorded during each hour.

On the first day of the campaign, 23 vaccination teams were deployed, followed by 27 teams (26 hand-catching and 1 net-catching) on each subsequent day. The mean vaccination rate was 17.3 dogs per team per day, with one vaccinator per team. Vaccination rates were highest on Days 1 and 2, at 21.0 and 22.1 dogs/team/day, respectively. However, rates declined on Days 3 and 4, with 14.7 and 11.3 dogs/team/day. This reduction is likely attributable to a combination of factors. These include worsening weather conditions such as heavy rainfall (**Fig 3**), a decreasing population of eligible unvaccinated dogs, and increased avoidance behaviour among dogs that had previously been captured or witnessed capture attempts.

Most vaccinated dogs were adults (n = 1,571; 88.2%), while 205 (11.5%) were puppies estimated to be under three months of age based on size and physical development. Among vaccinated dogs, 1,022 (57.4%) were male and 749 (42.1%) were female. Vaccination teams classified 1,539 (86.4%) dogs as unowned community dogs, and 236 (13.3%) as owned, with the majority observed to be free-roaming. Most vaccinated dogs appeared healthy (n = 1,473; 82.7%), with the most common conditions being skin disease or wounds (n = 221; 12.4%) and lameness (n = 81; 4.5%).

**Fig 4:**
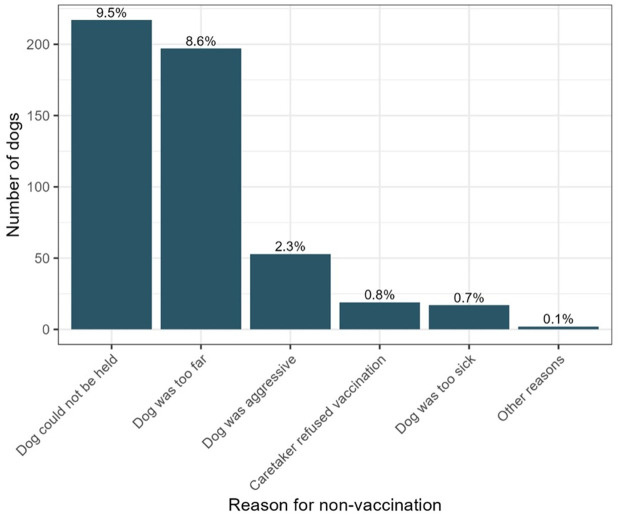
Reasons for non-vaccination in dogs that could not be vaccinated. Bar chart of dogs that were encountered by vaccination teams but could not be vaccinated. Percentage labels show each group as a proportion of all dogs encountered (including vaccinated dogs).

Of the 2,275 dogs encountered during the campaign, 494 (21.7%) were not vaccinated. The most common reasons for non-vaccination were inability to safely restrain the dog (n = 214; 43.3%) and dogs being too distant to approach (n = 194; 39.3%) (**Fig 4**). Demographic data were not collected for unvaccinated dogs.

During the campaign, three dogs exhibiting neurological signs consistent with suspected rabies were identified, all within the same camp block. Only one carcass was recovered for diagnostic testing, and results returned negative via lateral flow device (LFD) and direct fluorescent antibody (DFA) tests.

### Dog sight surveys

Post-vaccination dog sight surveys were conducted between 27 and 29 May 2025 across 34 of the 144 working zones (23.6%), covering 23 of the 26 sub-camps and adjacent peripheral areas. Over the three survey days, a total of 397 dogs were observed: 117 on Day 1, 84 on Day 2, and 196 on Day 3. Of dogs observed, 284 (71.5%) bore visible paint markings indicating vaccination, while 112 were unmarked and presumed unvaccinated. The proportion of marked dogs varied by day: 65.0% (76/117) on Day 1, 53.6% (45/84) on Day 2, and 83.2% (163/196) on Day 3. The overall proportion of marked dogs across all surveyed zones was 71.5% (95% CI: 66.8–75.9%).

### Community surveys

A total of 1,311 adult residents of the Kutupalong-Balukhali settlement participated in the community KAP survey. The mean age of respondents was 34.3 years (range: 18–82), and the majority were male (n = 1,035; 78.9%). To account for this overrepresentation, survey results are presented as both unadjusted estimates and estimates adjusted for gender using post-stratification weights based on the adult population gender distribution in the camps (**Table 1**). Adjusted estimates are used in this section to describe the findings.

**Table 1:** Gender distribution of adult survey respondents and post-stratification weights used to adjust for sample representativeness [25].

Gender	Respondents (N)	Survey sample (%)	Adult population (%)	Weight
**Female**	276	21.1	54.0	2.56
**Male**	1035	78.9	46.0	0.58

The KAP survey results (**Table 2**) revealed that awareness was limited among the survey population. Only 34.6% of respondents had heard of the disease, and fewer than half (41.2%) correctly identified dog bites as the primary route of transmission. Regarding appropriate post-bite care, only 25.9% of respondents indicated they would both wash the wound and seek medical care, in line with WHO guidelines [2]. In comparison, 51.1% would seek medical care only, 5.5% would wash the wound only, 15.9% were unsure of the correct response, and 1.6% stated they would take no action.

**Table 2:** Summary of responses from the community KAP survey.

Variable/ Question	Response category	All households (N = 1311) n (%) unadjusted	Adjusted % (by gender)
**Demographics**			
Age (years)	18-30	650 (49.6)	52.6
	31-40	346 (26.4)	26.1
	41-50	146 (11.1)	12.1
	51-60	118 (9.0)	6.6
	61-70	46 (3.5)	2.3
	70+	5 (0.4)	0.2
Household population	Mean persons/household	6.6	–
	Total survey population	8,624	–
**KAP questions**			
Q1: Have you heard of rabies?	Yes	503 (38.4)	34.6
	No	808 (61.6)	65.4
Q2: How is rabies transmitted?	Dog bite	593 (45.2)	41.2
	Do not know	714 (54.5)	58.6
	Other	4 (0.3)	0.2
Q3: What actions should be taken following a dog bite?	Wash wound, medical care	303 (23.1)	25.9
	Medical care only	687 (52.4)	51.1
	Wash wound only	95 (7.2)	5.5
	Nothing	22 (1.7)	1.6
	Do not know	205 (15.6)	15.9
Q4: Can dogs be vaccinated to prevent rabies?	Yes	740 (56.4)	56.2
	No	68 (5.2)	4.2
	Not sure	503 (38.4)	39.6
Q5: Are you happy for our team to vaccinate dogs in your block?	Yes	1249 (95.3)	95.6
	No	17 (1.3)	1.1
	Not sure	45 (3.4)	3.4
Q6: Are you or your family afraid of dogs in the camp?	A little afraid	469 (35.8)	31.4
	Very afraid	644 (49.1)	58.6
	Not afraid at all	198 (15.1)	10.0
Q7: Have you or a family member been bitten by a dog in the past 12 months?	Yes	114 (8.7)	8.7
	No	1197 (91.3)	91.3

When asked about dog vaccination, 56.2% of respondents were aware that dogs can be vaccinated against rabies, and 95.6% expressed support for the ongoing dog vaccination campaign in their community. Regarding fear of dogs, 90.0% of respondents reported some level of apprehension, with 58.6% stating they were “very afraid” and 31.4% reporting being “a little afraid” of dogs in the camp.

A total of 114 respondents (8.7%) reported that they or a household member had been bitten by a dog in the previous 12 months, suggesting that approximately 1 in 10 households experienced a dog bite annually. Based on an average household size of 6.6 persons, this corresponds to a minimum estimated bite incidence of 13.3 dog bites per 1,000 persons per year (95% CI: 11.0–15.9). Extrapolating this rate to the total camp population of 1,139,433, this suggests more than 15,000 dog bites occur annually in the Kutupalong-Balukhali settlement.

## Discussion

This study demonstrates that mass dog rabies vaccination campaigns are both possible and essential for safeguarding human and animal health in refugee settings. Vaccinating 1,781 of 2,275 dogs (78.3%) over four days in one of the world’s largest refugee settlements shows that structured campaigns can achieve high coverage, even in complex, resource-constrained environments. To our knowledge, this is the first structured mass dog vaccination in a refugee setting, providing practical lessons for rabies control in emergency and humanitarian contexts.

Central to the campaign’s success was the WVS-developed microplanning framework and operational technologies, previously applied in Bangladesh [28], India [23,24], and other countries worldwide. Coupled with real-time data collection via the WVS App, this framework enabled efficient planning and implementation, allowing teams to move systematically through the camps and achieve broad geospatial coverage. Post-vaccination dog sight surveys conducted in 23.6% of zones indicated that 71.5% of dogs observed were vaccinated. Interpretation was limited by the single-transect method, absence of identifiable data for unvaccinated dogs, and the dynamic nature of free-roaming dogs [28,29]. Nevertheless, these surveys provided actionable feedback to assess campaign outcomes and guide targeted mop-up efforts.

The community KAP survey revealed substantial gaps in rabies awareness and health-seeking behaviours. While 8.7% of households reported dog bites in the past year, only 25.9% of respondents (gender-adjusted) knew to wash wounds and seek medical care per WHO guidelines [2]. Moreover, only 34.6% had heard of rabies, and 41.2% recognised dog bites as the primary transmission route. These findings highlight the need to integrate education and awareness initiatives alongside vaccination programmes in refugee settings to improve health-seeking behaviour and reduce human rabies exposures and deaths.

Although operationally successful, the broader sustainability and cost-effectiveness of such campaigns require further evaluation. The Government of Bangladesh, in collaboration with local nonprofits and academic institutions, played a pivotal role in ensuring effective implementation and fostering local ownership. This engagement enhanced efficiency and underscores the importance of embedding dog vaccination programmes within national health frameworks adapted to humanitarian contexts. Strengthening partnerships, leveraging existing infrastructure, and aligning vaccination initiatives with broader public health priorities will be key to sustaining long-term impact.

Rabies remains a major threat in emergency and displacement settings. Countries such as Ukraine, Syria, and Sudan have reported resurging human rabies cases due to disrupted health systems and veterinary services, compounded by population displacement [30–32]. Vaccinating free-roaming dogs remains the most effective strategy to prevent human exposure. Integrating rabies control into humanitarian response strategies, in coordination with organisations such as MSF, UNICEF, and WHO, is essential to protect displaced populations from zoonotic threats and to ensure that rabies prevention is embedded within emergency health programmes [8].

In conclusion, this mass dog rabies vaccination campaign demonstrates that rabies control is achievable in refugee settings. The lessons learned offer a practical framework for integrating zoonotic disease control into humanitarian health programmes, reinforcing the right of all populations to be protected from preventable diseases and promoting equitable health for displaced communities.

## Supporting information

S1 Data Dog vaccination data. This CSV file contains the raw data collected during the mass dog vaccination campaign. Variables include GPS coordinates, vaccination action, ownership, age, sex, health condition, and, for dogs not vaccinated, the reason for non-vaccination.
(CSV)
